# Anti-Oxidant Activity of Gallotannin-Enriched Extract of Galla Rhois Can Associate with the Protection of the Cognitive Impairment through the Regulation of BDNF Signaling Pathway and Neuronal Cell Function in the Scopolamine-Treated ICR Mice

**DOI:** 10.3390/antiox8100450

**Published:** 2019-10-03

**Authors:** Ji Won Park, Ji Eun Kim, Mi Ju Kang, Hyeon Jun Choi, Su Ji Bae, Sou Hyun Kim, Young Suk Jung, Jin Tae Hong, Dae Youn Hwang

**Affiliations:** 1 Department of Biomaterials Science, College of Natural Resources & Life Science/Life and Industry Convergence Research Institute, Pusan National University, Miryang 50463, Korea; parkgeeone@naver.com (J.W.P.); prettyjiunx@naver.com (J.E.K.); sujibaebae@naver.com (S.J.B.); 2 College of Pharmacy, Pusan National University, Busan 46241, Korea; hyunie0731@naver.com (S.H.K.);; 3 College of Pharmacy, Chungbuk National University, Chungju 28160, Korea; jinthong@chungbuk.ac.kr

**Keywords:** galla rhois, cognitive impairment, scopolamine, acetylcholinesterase, BDNF

## Abstract

The antibacterial, anti-inflammatory, anti-metastatic/anti-invasion activities and laxative activity of *Galla Rhois* (GR) are well-known, although the neuropreservation effects of their extracts are still to be elucidated. To investigate the novel therapeutic effects and molecular mechanism of GR on alleviation of cognitive impairment, two different dosages of gallotannin-enriched GR (GEGR) were administered to Korl:ICR mice for three weeks, and to induce memory impairment, scopolamine (SP) was administered during the last seven days of the GEGR treatment period. GEGR showed the high level of the free radical scavenging activity to DPPH and suppressive activity to reactive oxygen species (ROS) in B35 cells as well as enhanced SOD and CAT activity in brains of the SP-induced model. Latency time for memory impairment assessed by the passive avoidance test significantly protected in the SP+GEGR treated group as compared to the SP+Vehicle treated group. Moreover, similar protective effects were observed on the secretion of BDNF in SP+GEGR treated mice. The expression of TrkB receptor, and phosphorylation of PI3K on the TrkB receptor signaling pathway were dramatically protected in the SP-induced model after GEGR treatment, whereas the expression of p75^NTR^ receptor, the phosphorylation of JNK, and expression of Bax/Bcl-2 on the p75^NTR^ receptor signaling pathway was significantly protected in the same group. Furthermore, the GEGR treated SP-induced model showed decreased number of dead neural cells and suppressed acetylcholine esterase (AChE) activity and inhibited inflammatory responses. Taken together, these results indicate that the anti-oxidant activity of GEGR contributes to improving the neuronal cell function and survival during cognitive impairment in the SP-induced model through regulation of BDNF secretion and their receptor signaling pathway.

## 1. Introduction

Oxidative stress leading to DNA oxidation, protein oxidation, lipid oxidation, and glycoxidation induces an imbalance between the production of reactive oxygen species (ROS) and the antioxidant defense system [1]. This stress plays a key role in the initiation and progression of Alzheimer’s disease (AD), along with resultant neuronal damage [2]. Oxidative stress also stimulates the production of amyloid beta (Aβ) peptides and the phosphorylation of tau proteins [3]. Furthermore, enhancement of Aβ accumulation results in increased oxidative stress and mitochondrial dysfunction via inhibition of ATP production and increase of reactive oxygen species (ROS) generation [3,4,5]. Hence, antioxidant treatment is considered an effective strategy for inhibiting the initiation and progression of AD.

Among the various antioxidants, tannin and tannin-related plant extracts have received great attention, since recent studies have demonstrated the tight correlation between antioxidant treatments and symptomatic relief of AD symptoms [6]. The aggregation and production of Aβ are significantly inhibited by exposure to gallotannin and tannic acid in in vitro studies and the PSAPP mouse model presenting AD-like pathology [7,8]. Moreover, similar inhibitory effects on Aβ plaque deposition and β-amyloid fibrils (fAβ) formation have been reported in APP/PS1 and PSAPP Tg mice treated with pomegranate polyphenols and tannic acid [9,10]. Gallotannin derived from *Cornus officinalis* and tannic acid significantly inhibit the beta-secretase (BACE1), acetylcholinesterase (AChE), and butyrylcholinesterase (BChE) activities [8,11]. Furthermore, the inhibitory effects of tannic acid have been verified during the in vitro aggregation analysis of tau peptide R3 and electron microscopy study of human full-length tau protein (tau441) [12,13]. However, the neuroprotective effects and the molecular mechanism of gallotannin-enriched *Galla Rhois* (GEGR) in the scopolamine (SP)-induced memory impairment model are not fully investigated, although gallotannin, gallic acid, and methyl gallate are identified as the major components of GEGR [14].

Meanwhile, brain-derived neurotrophic factor (BDNF) is well known as an important molecule during synapse development and plasticity [15]. This molecule stimulates the neural regeneration as well as enhanced the cognitive and memory function in mammalian system through the interaction with TrkB [16]. However, the precursor of BDNF (pro-BDNF) exhibits opposite biological function of BDNF via the interaction mainly with p75^NTR^ [16,17]. Especially, BDNF has the ability to cross blood-brain barrier (BBB) and be stably maintained in blood up to 60 min after intravenous injection [18]. Therefore, BDNF has been considered as one of the treatment strategies for cognitive-related disease such as aging and AD [19].

The present study evaluated the possibility of developing a new natural medicine by investigating cognitive impairment, cell function and survival, and BDNF regulation during the neuroprotective effects of GEGR in an SP-induced AD model. The present study provides the first scientific evidence that GEGR is a tannin-containing natural product that successfully induces neuroprotective effects in the AD animal model through the regulation of neuronal cells function and BDNF signaling pathway.

## 2. Materials and Methods

### 2.1. Purification of GEGR

The GEGR samples were prepared as described previously [14,20]. In October 2013, samples of dry GR were collected from plantations in the Hongcheon area of Korea. Voucher specimens of GR (WPC-14-001) were deposited in the Functional Materials Bank at the Pusan National University–Wellbeing RIS Center. Collected specimens were dried further in a hot-air drying machine (JSR, Seoul, Korea) at 60 °C and then powdered by using an electric blender. A water extract of GR was obtained by treating a 1:10 ratio GR powder: Water mixture for 9 h at 90 °C in a circulating extractor (IKA Labortechnik, Staufen, Germany). The GR extract was passed through a 0.4 µm filter and then concentrated via vacuum evaporation and lyophilization in an IKA circulating extraction system (IKA Labortechnik, Staufen, Germany). The obtained GEGR extract powder was dissolved in distilled water (dH_2_O) to a final concentration of 1 mg/mL, and further diluted with 1× phosphate buffered saline (PBS) to the concentration required.

### 2.2. Free Radical Scavenging Activity of GEGR

The 2,2-diphenyl-1-picrylhydrazyl (DPPH) radical scavenging activity level was determined using a previously described method [21,22]. Briefly, the powdered GEGR was dissolved in 50% EtOH (100 μL) to obtain 12 different GEGR concentrations (1 to 2000 μg/mL), which were then mixed with 100 μL of 0.1 mM DPPH (Sigma-Aldrich Co., St. Louis, MO, USA) in a 95% ethanol solution or with 100 μL of 95% ethanol solution (control). The mixtures were then incubated at room temperature for 30 min. A VersaMax plate reader (Molecular Devices, Sunnyvale, CA, USA) was used to determine the reaction mixture absorbance at 517 nm. The DPPH radical scavenging activity of GEGR is expressed as the percent decrease in absorbance relative to the control. The IC_50_ value indicates the substrate concentration that produces a 50% decrease in DPPH radical scavenging activity.

### 2.3. ROS Levels Analyses in B35 Cells and Brain Tissues

Intracellular ROS levels were measured in B35 cells (neural neuroblast cells that originate from the neuroblastoma of BDIX rats) by staining with 2′,7′-dichlorofluorescein diacetate (DCFH-DA) (Sigma-Aldrich Co.), as previously described [22]. Briefly, B35 cells were seeded at 5 × 10^5^ cells/2 mL in six-well plates and incubated with 100 μg/mL of GEGR for 1 h at 37 °C. After washing with 1× PBS, the cells were incubated with H_2_O_2_ (100 μM; Junsei Chemical Co.) for 24 h. Next, the stimulated cells along with 25 μM DCFH-DA were incubated for 30 min at 37 °C. Finally, the cells were washed twice with 1× PBS, and the presence of green fluorescence was observed under a fluorescence microscope (200× magnification; Eclipse TX100, Nikon, Tokyo, Japan).

ROS level in the brain cortex tissue was assessed by employing a proprietary quenched fluorogenic probe and the reagents provided in the OxiSelect™ In Vitro ROS/RNS Assay Kit (Cell Biolabs Inc., San Diego, CA, USA). Briefly, the brain cortex tissue samples (100 mg) were homogenized in 600 μL of 1× PBS using a glass homogenizer. Each sample and standards (50 μL) were mixed with Catalyst (50 μL) in 96-well plate for 5 min. DCFH solution (100 μL) were added into this mixture and incubated under the light protection for 15–45 min at room temperature. The final fluorescence of each well were measured at 480 nm excitation/530 nm emission.

### 2.4. Design of Animal Experiment

The Pusan National University—Institutional Animal Care and Use Committee reviewed and approved the animal protocol used in this study (approval number; PNU-2018-1809). All mice were handled at the Pusan National University—Laboratory Animal Resources Center (accredited by the Korea Food and Drug Administration [unit 000231] and the Association for Assessment and Accreditation of Laboratory Animal Care International [unit 001525]). Male Korl:ICR mice (six-weeks-old) were kindly provided by the Department of Laboratory Animal Resources of the National Institute of Food and Drug Safety Evaluation (Cheongju, Korea). Water and a standard irradiated chow diet (Samtako BioKorea Co., Osan, Korea) were provided *ad libitum* to all animals throughout the experimental period. Furthermore, all mice were maintained under specific pathogen-free (SPF) conditions at 23 ± 2 °C, 50 ± 10% relative humidity, and a strict light:dark cycle (lights on at 08:00 h and off at 20:00 h) during the experiments.

The Korl:ICR mice (*n* = 60) were first divided into No (*n* = 12) and SP treated (*n* = 48) groups. The No group was not exposed to any experimental treatment; the SP-treated group was divided into four subgroups (*n* = 12 each): SP+Vehicle treated, SP+donepezil (DP) treated, SP+Low dose GEGR (LGEGR) treated, and SP+High dose GEGR (HGEGR) treated groups. Briefly, the SP+DP, SP+LGEGR, and SP+HGEGR treated groups were administered DP (2 mg/Kg, p.o.) or GEGR (100 mg/Kg or 200 mg/Kg, p.o.) daily for 21 days, while the SP+Vehicle treated group was administered 1× PBS (p.o.). From the 17th to the 21st day, a daily dose of SP (Sigma-Aldrich Co.; 1 mg/kg body weight) was injected intraperitoneally into members of four of the groups (SP+Vehicle, SP+DP, SP+LGEGR, and SP+HGEGR). At 1 h after the final treatment, mice in all groups underwent passive avoidance testing. The mice were then euthanatized in a chamber filled with CO_2_ gas and brain samples were subsequently collected.

### 2.5. Passive Avoidance Test

Passive avoidance testing of the variously treated Korl:ICR mice was conducted as previously described [23]. The passive avoidance test apparatus (490 mm × 250 mm × 300 mm; Daejong Inc., Seoul, Korea) consisted of illuminated and dark compartments separated by an acryl plate in which there was a small passage. During the learning stage, a mouse is placed in the illuminated compartment and the time spent in the illuminated compartment until the mouse steps through the open passage into the dark compartment is defined as the latency period. At three s after entering the dark compartment, a shock (0.5 mA, 200 V) is delivered to the feet of the mouse via a floor electrical grid in the dark compartment. The mouse can escape the shock by re-entering the illuminated (safe) compartment. During the learning stage, these acquisition trials were conducted three times in the first day and once without shock in the second day; a mouse was judged to have learned to avoid the shock when the latency period was 300 s. To accomplish this, latency was measured for up to 300 s without delivering a foot shock. A mouse was considered to have retained avoidance memory if it remained in the illuminated compartment for 100 s.

### 2.6. Determination of AChE Activity

The level of AChE activity was determined by using an Acetylcholinesterase Assay Kit (Abcam, Cambridge, UK) in accord with the manufacturer’s protocols. Briefly, the brain cortex from each mouse was homogenized in PRO-PREP protein extraction solution (1.0 mM PMSF, 1.0 mM EDTA, 1.0 μM pepstatin, 1.0 μM leupeptin, and 1.0 μM aprotinin; iNtRON Biotechnology Inc., Seoul, Korea); prior to AChE analysis, the homogenate was stored at −70 °C. For analysis, the homogenate sample (or standards) and the AChE reaction mixture were incubated for 20 min at room temperature in a 96-well plate protected from light. Color alteration within the plate wells was determined by using a Vmax plate reader (Molecular Devices, Sunnyvale, CA, USA) at 405 nm.

### 2.7. Analysis of Superoxide Dismutase (SOD) Activity

SOD activity in the brain cortex tissue was assessed by applying a calorimetric assay and the reagents provided in the SOD assay kit (Dojindo Molecular Technologies Inc., Rockville, MD, USA). Initially, the brain cortex tissue samples (100 mg) were homogenized in 600 μL of sucrose buffer (0.25 M sucrose, 10 mM HEPES, 1 mM EDTA, pH 7.4) using a glass homogenizer. Lysate was harvested by centrifugation of the homogenate at 10,000× *g* for 60 min, then stored at −70 °C until assayed. To determine the SOD activity level, the sample lysate was diluted with the dilution buffer or saline to the following ratios: 1, 1/5, 1/52, 1/53, 1/54, 1/55, and 1/56. Aliquots of each sample solution (25 μL) were subsequently placed in individual wells of a 96-well plate along with 200 μL of the WST-1 working solution. In addition, 20 μL of the enzyme working solution was added to each well, followed by thorough mixing. The enzyme reaction was induced by incubating the prepared plate at 37 °C for 20 min, after which the absorbance of each well was measured at 450 nm using a spectrophotometer. Finally, the level of SOD activity was calculated directly by using the equation:

SOD activity (inhibition rate %) = [(A blank 1 − A blank 3) − (A sample − A blank 2)]/(A blank 1 − A blank 3) × 100,

where, A blank 1, 2, and 3 indicate the absorbances of blanks 1, 2, and 3, respectively, and A sample is the absorbance of the sample.

### 2.8. Analysis of Catalase (CAT) Activity

CAT activity in the brain cortex tissue was assessed using the EZ-Catalase assay kit (DoGenBio, Seoul, Korea). After the preparation of brain tissue homogenates, a volume of 25 μL of brain sample was mixed with 25 μL of H_2_O_2_ solution. Subsequently, Oxi-Probe/Horseradish peroxidase solution (100 μM and 0.4 Units/mL dissolved in 1× reaction buffer, pH 7.5) was added to each well and incubated at room temperature for 30 min in the dark. The activity was monitored by measuring fluorescent intensity using a Glomax microplate reader (Promega, Madison, WI, USA). Results were corrected by protein concentration determined with BCA protein assay (Thermo Scientific, Rockford, IL, USA), and expressed as units per mg of brain protein. One unit of catalase was defined as 1 μmol H_2_O_2_ decomposed per min.

### 2.9. Quantitative Real Time-PCR Analysis

Frozen brain tissue was chopped with scissors and homogenized in RNA Bee solution (Tet-Test Inc., Friendswood, TX, USA). Total RNA molecules were isolated by centrifugation at 10,000× *g* for 15 min, and the total RNA concentration was measured by UV spectroscopy. The complementary DNA (cDNA) was synthesized by Invitrogen Superscript II reverse transcriptase (Thermo Scientific, Wilmington, DE, USA). Quantitative PCR (qPCR) was performed with the cDNA template (1 μL) and 2x Power SYBR Green (6 μL; Toyobo Life Science, Osaka, Japan) containing specific primers. The primer sequences used for target gene expression identification were as follows: SOD, sense 5′-GTG AAC CAG TTG TGT TGT CAG GAC-3′ and anti-sense 5′-GAT GGA ATG CTC TCC TGA GAG TGA GAT C-3′; COX-2, sense 5′-CAG GTC ATT GGT GGA GAG GTG TAT C-3′ and anti-sense 5′-CCA GGA GGA TGG AGT TGT TGT AGA G-3′; IL-1β, sense 5′- CTG TCC TGA TGA GAG CAT CCA GCT TC-3′ and anti-sense 5′-GTT GCT TGG TTC TCC TTG TAC AAA GCT C-3′; IL-10, sense 5′- CTC TTA CTG ACT GGC ATG AGG ATC AG-3′ and anti-sense 5′-CTA TGC AGT TGA TGA AGA TGT CAA ATT C-3′; β-actin, sense 5′- TGG AAT CCT GTG GCA TCC ATG AAA C -3′ and anti-sense 5′- TAA AAC GCA GCT CAG TAA CAG TCC G -3′. qPCR was performed for 40 cycles using the following parameters: Denaturation at 95 °C for 15 s, followed by annealing and extension at 70 °C for 60 s. Fluorescence intensity was measured at the end of the extension phase of each cycle. The threshold value for the fluorescence intensities of all samples was set manually. The reaction cycle at which the PCR products exceeded this fluorescence intensity threshold during the exponential phase of PCR amplification was considered as the threshold cycle (Cq). Expression of the target gene was quantified relative to that of the housekeeping gene β-actin, based on comparison of the Cqs at a constant fluorescence intensity, as per the Livak and Schmittgen’s method [24].

### 2.10. Western Blot

Protein homogenates from mouse brain cortex tissue and B35 cells were prepared by using a protein extraction (pro-prep) solution (iNtRON Biotechnology, Burlington, MA, USA). The total proteins thus obtained were separated by performing 8–12% sodium dodecyl sulfate-polyacrylamide gel electrophoresis (SDS-PAGE) for 2 h, after which the SDS-PAGE-resolved proteins were transferred (2 h at 40 V) to nitrocellulose membranes. Individual membranes were incubated overnight at 4 °C with the following primary antibodies: Anti-BDNF (Santa Cruz Biotechnology), anti-TrkB (Abcam), anti-PI3K (Cell Signaling Technology Inc.), anti-p-PI3K (Cell Signaling Technology Inc.), anti-ERK (Santa Cruz Biotechnology), anti-p-ERK (Santa Cruz Biotechnology), anti-p-75^NTR^ (Cell Signaling Technology Inc.), anti-JNK (Cell Signaling Technology Inc.), anti-p-JNK (Cell Signaling Technology Inc.), anti-Bax (Abcam), anti-Bcl-2 (Invitrogen), anti-SOD (Abcam), anti-Nrf2 (Abcam), anti-p-Nrf2 (Invitrogen), or anti-actin antibody (Sigma-Aldrich Co.). Next, the membranes were washed (washing buffer comprised of 137 mM NaCl, 2.7 mM KCl, 10 mM Na_2_HPO_4_, and 0.05% Tween 20) and then incubated at room temperature for 1 h with horseradish peroxidase (HRP)-conjugated goat anti-rabbit IgG (Invitrogen, Carlsbad, CA, USA) diluted 1:1000. The membrane blots were developed by using Amersham ECL Select Western Blotting detection reagent (GE Healthcare, Little Chalfont, UK).

### 2.11. Histological Analysis

Brain perfusion and Nissl staining were performed as described previously [22]. Briefly, mice were anesthetized by an intraperitoneal Alfaxan injection (80 mg/kg body weight) before undergoing transcardial perfusion with 1× PBS followed by 4% formaldehyde to effectively remove blood and fix the brain tissue. Subsequently, the brain of each mouse was isolated from the skull, fixed overnight in formaldehyde, and then embedded in paraffin and sectioned for Nissl staining. Slides bearing brain sections (10 μm) underwent Nissl staining with the 0.1% cresyl violet acetate stain solution for 8 min, followed by washing with dH_2_O. For each slid, the surviving neurons were enumerated using the modified method described previously [22]. To determine the degree of neuronal loss in the hippocampus, tissue sections of both hemispheres from 5–7 mice/group were also stained with hematoxylin and eosin. The surviving pyramidal neurons in cornu ammonis 1 (CA1) and CA3 regions were counted (per 500 μm; 200× magnification) bilaterally and averaged.

### 2.12. Statistical Analysis

Statistical analyses were performed by using the tests within SPSS software version 10.10 (SPSS, Inc. Chicago, IL, USA). One-way analysis of variance, followed by Tukey’s post hoc test, was performed to identify significant differences between the Vehicle and GEGR- or DP-treated groups, and between the No and SP-treated groups. Data showing no normal distribution were analyzed in Kruskal-Wallis and Mann-Whitney U test. All values presented are means ± standard deviations. A *p* value < 0.05 is considered to indicate a statistically significant difference.

## 3. Results

### 3.1. Anti-Oxidant Activity and BDNF Recovery Effects of GEGR in B35 Cells

**Figure 1:**
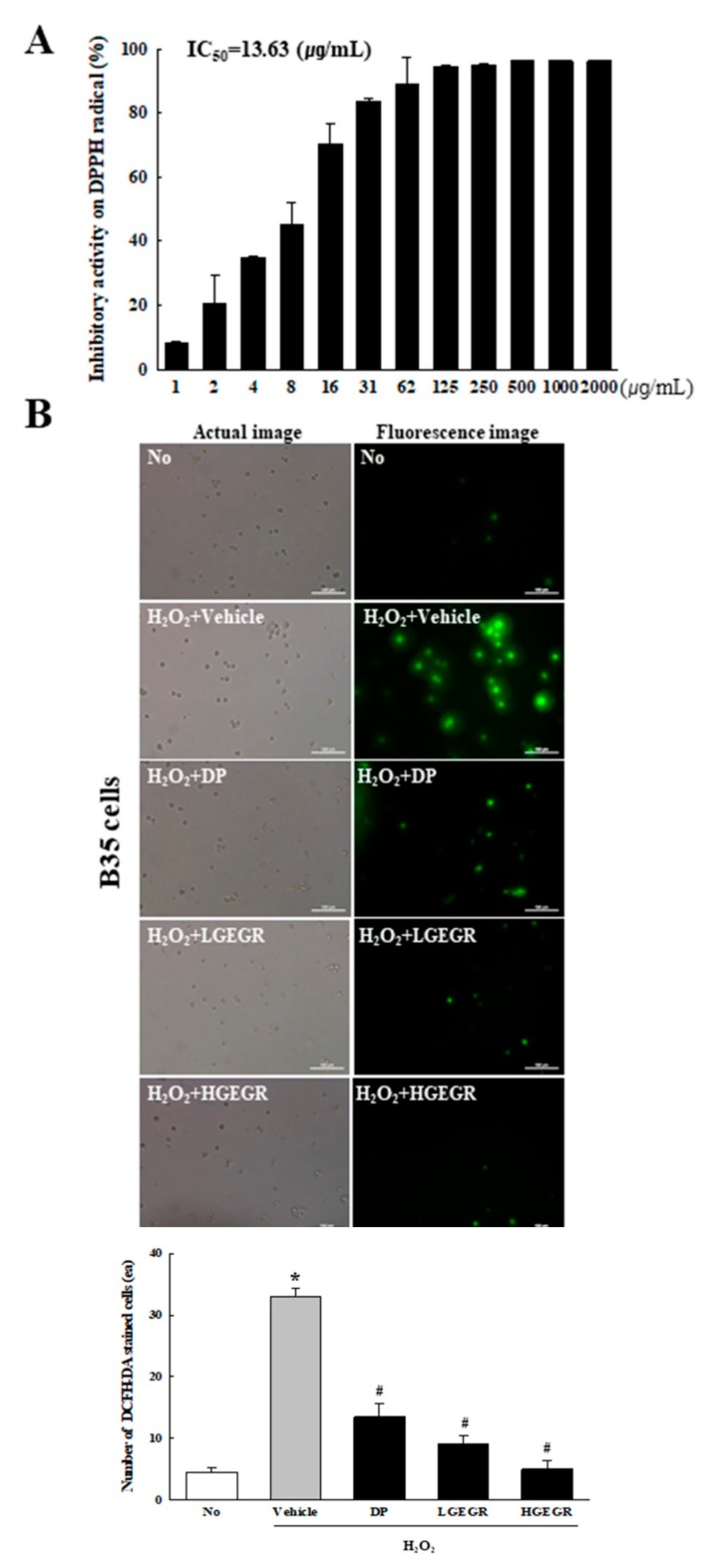

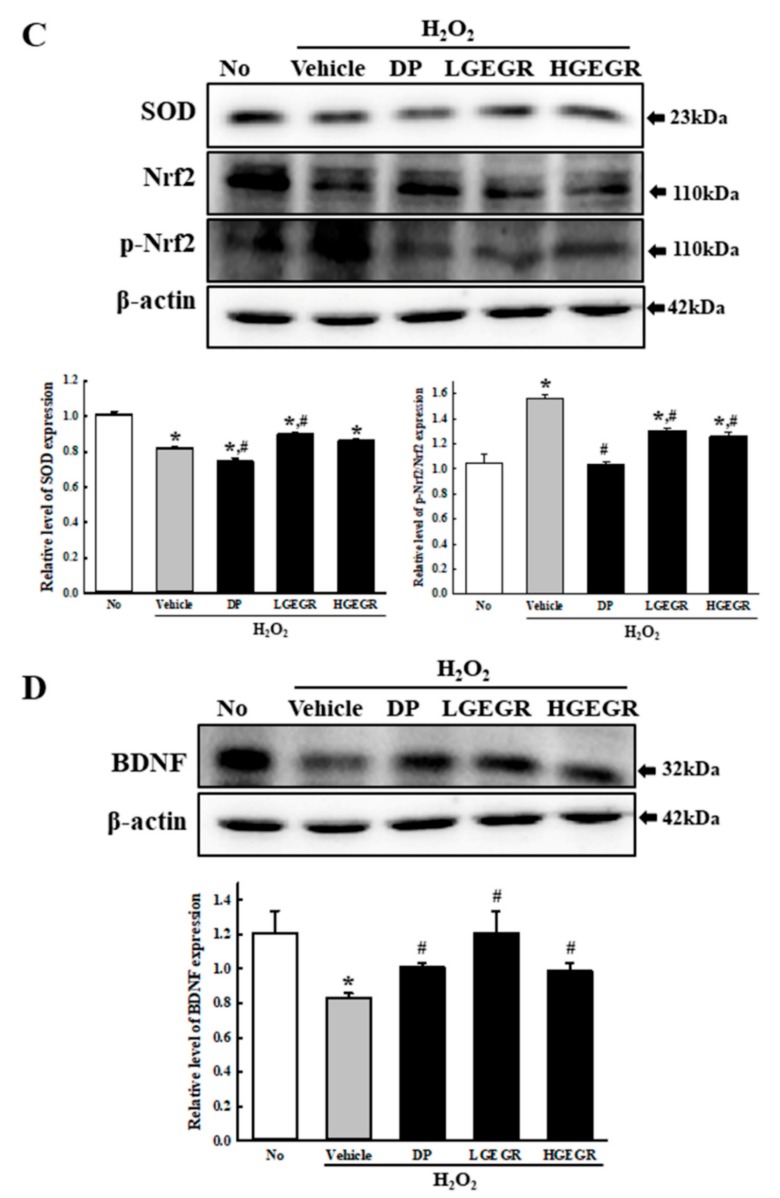
Anti-oxidant activity and BDNF secretion activity of GEGR in vitro. (**A**) Free radical scavenging activity of GEGR. DPPH radical scavenging activity was assayed in a mixture containing 0.1 mM DPPH within a range of GEGR concentrations from 1 to 2000 μg/mL. (**B**) Determination of intracellular ROS production. After 2’,7’-dichlorofluorescein diacetate (DCFH-DA) treatment, green fluorescence in cells of each subset group was observed using a fluorescent microscope. B35 cells in each square of a 200× magnification image (left column) were also examined under 400× magnification (right column). (**C**) Detection of superoxide dismutase (SOD), Nrf2, and p-Nrf2 protein. Total tissue homogenates were prepared from brains of scopolamine (SP)-injected mice treated with Vehicle or GEGR as described in Materials and Methods. Three proteins were detected with specific antibody and quantified using an imaging densitometer. (**D**) Detection of brain-derived neurotrophic factor (BDNF) protein. Total tissue homogenates were prepared from brains of SP-injected mice treated with Vehicle or GEGR as described in Materials and Methods. BDNF protein was detected with specific antibody and quantified using an imaging densitometer. Three samples were assayed in duplicate by western blotting. * *p* < 0.05 compared with the No treated group. ^#^ *p* < 0.05 compared with the SP+Vehicle treated group. Abbreviation: DPPH, 2,2-diphenyl-1-picrylhydrazyl radical; IC_50_, half maximum inhibitory concentration; GEGR, gallotannin-enriched extract of Galla Rhois; ROS, reactive oxygen species.

To confirm the anti-oxidant activity and BDNF recovery effects of GEGR before analyzing the molecular mechanism on alleviation of cognitive impairment, we determined the free radical scavenging activity, the inhibitory effects against ROS production and BDNF secretion in B35 cells. The inhibitory activity against DPPH radical gradually enhanced at concentrations of 1–125 μg/mL of GEGR, with an IC_50_ value of GEGR determined at 13.63 μg/mL (Figure 1A). A similar anti-oxidant activity of GEGR was observed in the inhibitory effects against H_2_O_2_-induced ROS production, wherein the ROS production was remarkably decreased in GEGR+H_2_O_2_ treated B35 cells without any significant changes in their morphology (Figure 1B). Moreover, some significant protection on the expression of SOD and p-Nrf2 were detected in the H_2_O_2_+GEGR treated group (Figure 1C). Furthermore, the decreased BDNF expression in the H_2_O_2_+Vehicle treated cells was significantly protected in the H_2_O_2_+GEGR treated group (Figure 1D). These results indicate the high anti-oxidant activity and neuroprotective effects of GEGR, and the likely association to its neuroprotective effects in the animal model for AD.

### 3.2. Anti-Oxidant Activity of GEGR in SP-Induced Memory Impairment Model

**Figure 2:**
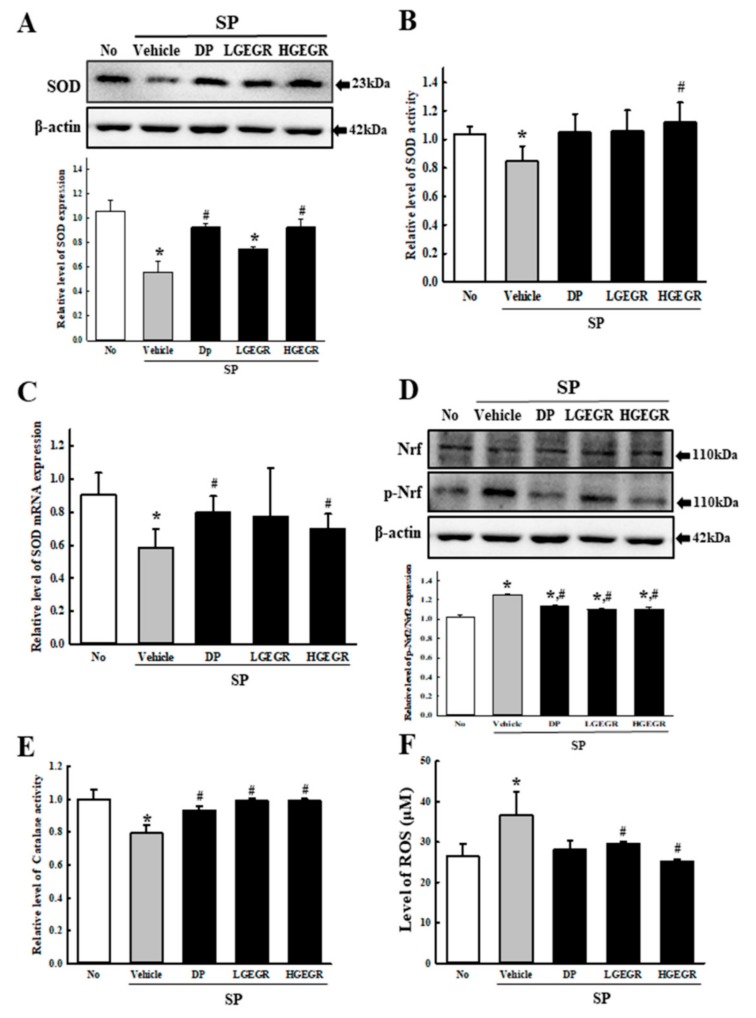
SOD activity, catalase (CAT) activity, and ROS concentration in brain of SP+GEGR treated mice. (**A**) Total tissue homogenates were prepared from brains of SP-injected mice treated with Vehicle or GEGR as described in Materials and Methods. SOD protein were detected from total protein (50 μg per sample) using specific protein. (**B**) The SOD activity level was measured in homogenates of brain tissue collected from each subset group as described in Materials and Methods. One SOD unit is defined as the amount of the enzyme in 20 μL of the sample solution that inhibits by 50% the reduction reaction of water-soluble tetrazolium salt-1 (WST-1) with superoxide anion. (**C**) The levels of SOD transcripts in the total messenger RNA (mRNA) of brain were measured by quantitative real-time (RT)-PCR analyses using specific primers. The mRNA level of SOD gene was calculated, based on the intensity of actin as an endogenous control. (**D**) Total tissue homogenates were prepared from brains of SP-injected mice treated with Vehicle or GEGR as described in Materials and Methods. Nrf2 and p-Nrf2 protein were detected from total protein (50 μg per sample) using specific protein. (**E**) The CAT activity was measured in homogenates of brain tissue collected from each subset group as described in Materials and Methods. Catalase 1 unit is defined as the amount of enzyme required to decompose 1 μmole of H_2_O_2_ per min at pH 7.0 and 25 °C. (**F**) ROS level was measured in homogenates of brain tissue collected from each subset group as described in Materials and Methods. This assay kit has a detection sensitivity limit of 10 pM for 2’,7’-dichlorofluorescein (DCF) and 40 nM for H_2_O_2_ respectively. Three samples were assayed in duplicate by western blotting. Data presented are means ± SD of duplicates. * *p* < 0.05 compared with the No group. ^#^ *p* < 0.05 compared with the SP+Vehicle treated group. Abbreviation: DP, donepezil; GEGR, gallotannin-enriched extract of Galla Rhois; SOD, superoxide dismutase; SP, scopolamine.

To investigate whether the anti-oxidant activity of GEGR in vitro can be completely reflected into the memory impairment model, the mRNA, protein and activity level of SOD, Nrf2 expression, CAT activity, and ROS concentration were determined in the brain tissue of the SP-induced memory impairment model. A similar alteration pattern was observed on the mRNA level, protein level, and enzyme activity of SOD. These levels were lower in SP+Vehicle treated group than the No treated group. After the treatment of DP and GEGR, these levels were remarkably enhanced with dose-dependent manner (Figure 2A–C). Moreover, these alterations on the level of SOD-related factors was reflected in a control of transcription level. The expression level of p-Nrf2, a transcription factor for the regulation of antioxidant enzyme, was increased in the cortex tissue of the SP+GEGR treated model although these of Nrf2 were constantly maintained (Figure 2D). Furthermore, an alteration on the CAT activity and ROS concentration were very similar with the regulation pattern for SOD and p-Nrf2 expression (Figure 2E,F). These results suggest that the anti-oxidant activity of GEGR detected in B35 cells may successfully exhibit in the animal model for AD.

### 3.3. Protective Effects of GEGR against Memory Impairment

**Figure 3:**
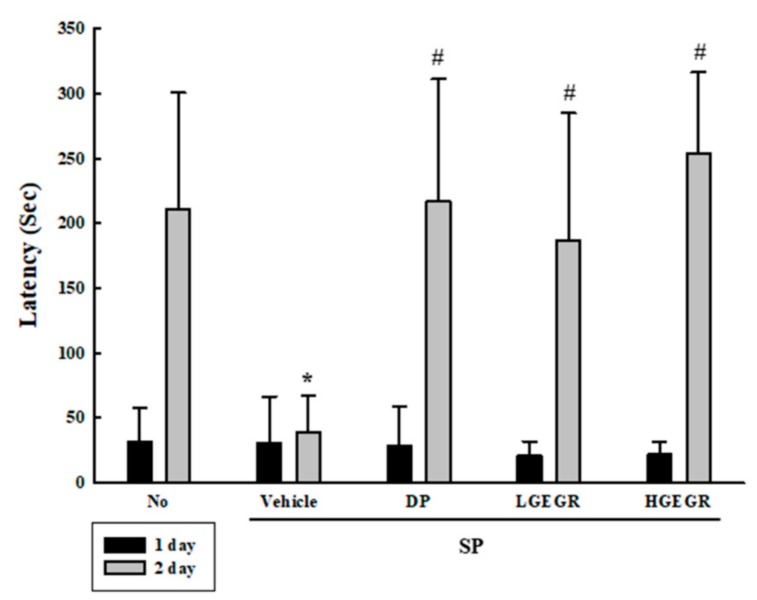
Alteration of cognition in SP+GEGR treated mice. A passive avoidance test was used to determine step-through latency for 300 s. Behavioral changes in mice were measured after SP injection. Ten to twelve mice per group underwent cognitive defect testing. Data represent the means ± SD of duplicates and analyzed by Kruskal-Wallis and Mann-Whitney U test. * *p* < 0.05 compared with the No treated group. ^#^ *p* < 0.05 compared with the SP+Vehicle treated group. Abbreviation: DP, donepezil; GEGR, gallotannin-enriched extract of Galla Rhois; SP, scopolamine.

We investigated whether the anti-oxidant activity of GEGR is accompanied with the protective effects against long-term memory defects. To achieve this, the exploratory preference was measured in SP+GEGR treated mice using passive avoidance tests. The latency time was short in mice of all groups during the training session, since these mice entered the dark box immediately after being placed in the illuminated box. However, this time was remarkably enhanced in the No treated group during the retention trial as compared to the acquisition trial, although no difference was observed in the SP+Vehicle group between each trial. Furthermore, the levels were significantly enhanced in the SP+GEGR treated group as compared to the SP+Vehicle group during the retention trial (Figure 3). These results, therefore, indicate that GEGR pretreatment protects the long-term memory defect induced by SP injection.

### 3.4. Effect of GEGR on BDNF Secretion and Their Receptor Signaling Pathway

**Figure 4:**
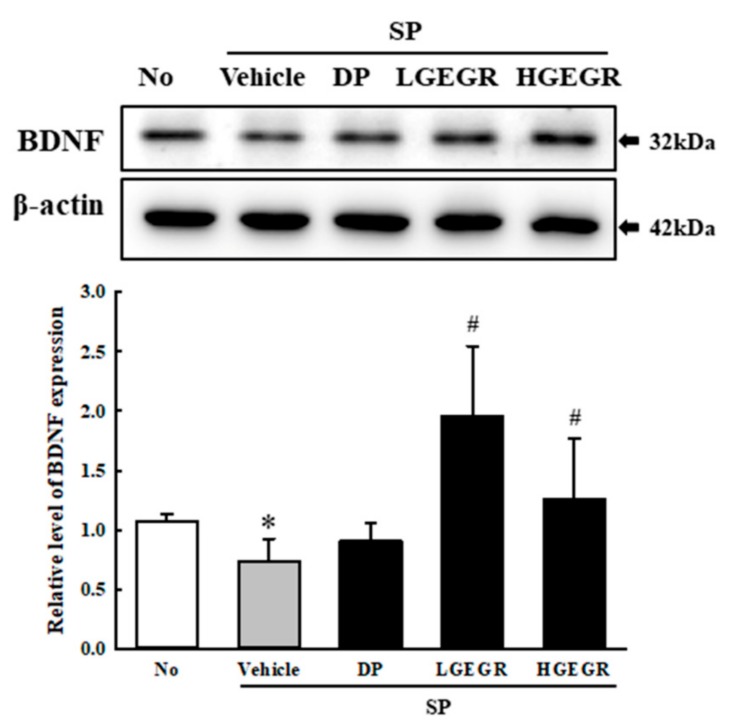
Secretion level of BDNF protein. Total tissue homogenates were prepared from brains of SP-injected mice treated with Vehicle or GEGR as described in Materials and Methods. Total protein (50 μg per sample) was immunoblotted with antibodies for the BDNF protein. After determining the individual band intensity level using an imaging densitometer, the relative levels of the proteins were calculated based on the intensity of actin. Three samples were assayed in duplicate by western blotting. Data are reported as mean ± SD values. * *p* < 0.05 compared with the No treated group. ^#^ *p* < 0.05 compared with the SP+Vehicle treated group. Abbreviation: DP, donepezil; GEGR, gallotannin-enriched extract of Galla Rhois; SP, scopolamine.

**Figure 5:**
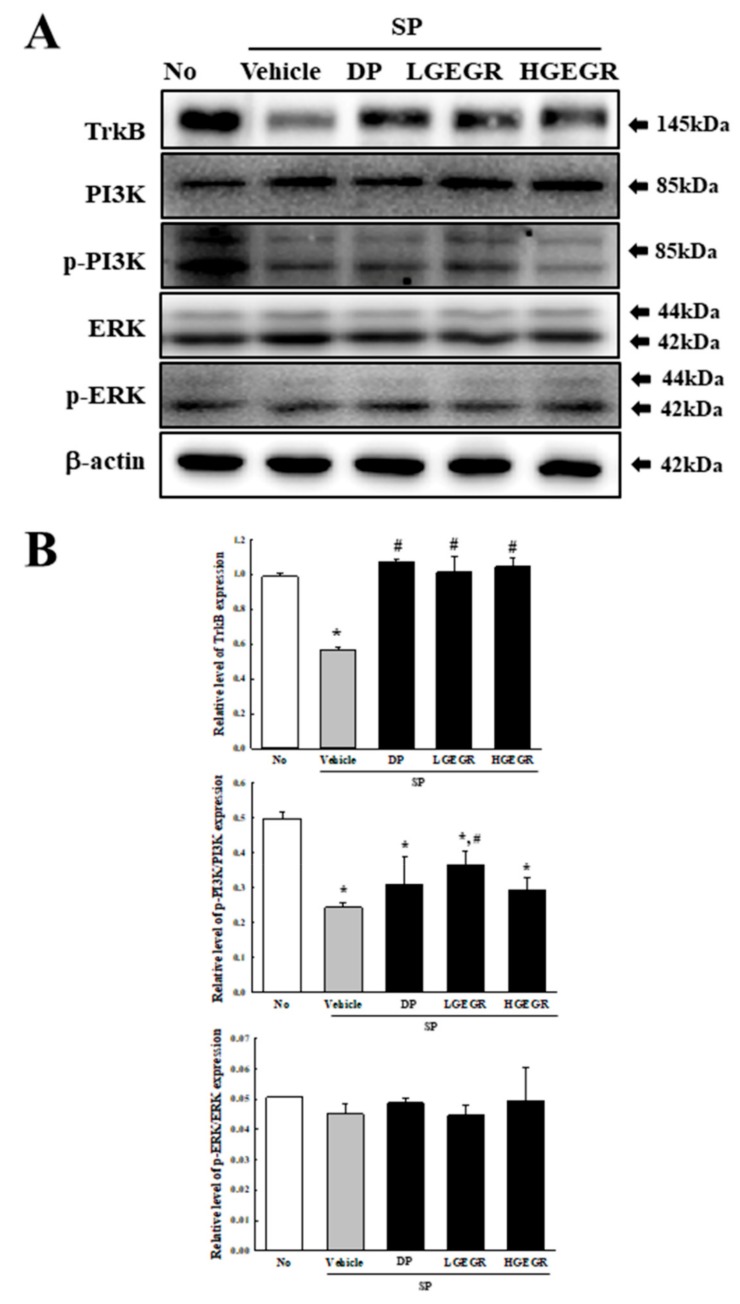
Alteration of the tropomyosin receptor kinase B (TrkB) receptor signaling pathway. Total tissue homogenates were prepared from the brain of Vehicle or GEGR treated SP-injected as described in Materials and Methods. Total protein (50 μg per sample) was immunoblotted with TrkB, p-TrkB, PI3K, p-PI3K, ERK, p-ERK, or β-actin antibodies. The intensity of each band was determined by using an imaging densitometer, and the relative levels of the proteins were based on the intensity of actin. Three samples were assayed in duplicate by western blotting. Data are reported as mean ± SD values. * *p* < 0.05 compared with the No treated group. ^#^ *p* < 0.05 compared with the SP+Vehicle treated group. Abbreviation: DP, donepezil; GEGR, gallotannin-enriched extract of Galla Rhois; SP, scopolamine.

**Figure 6:**
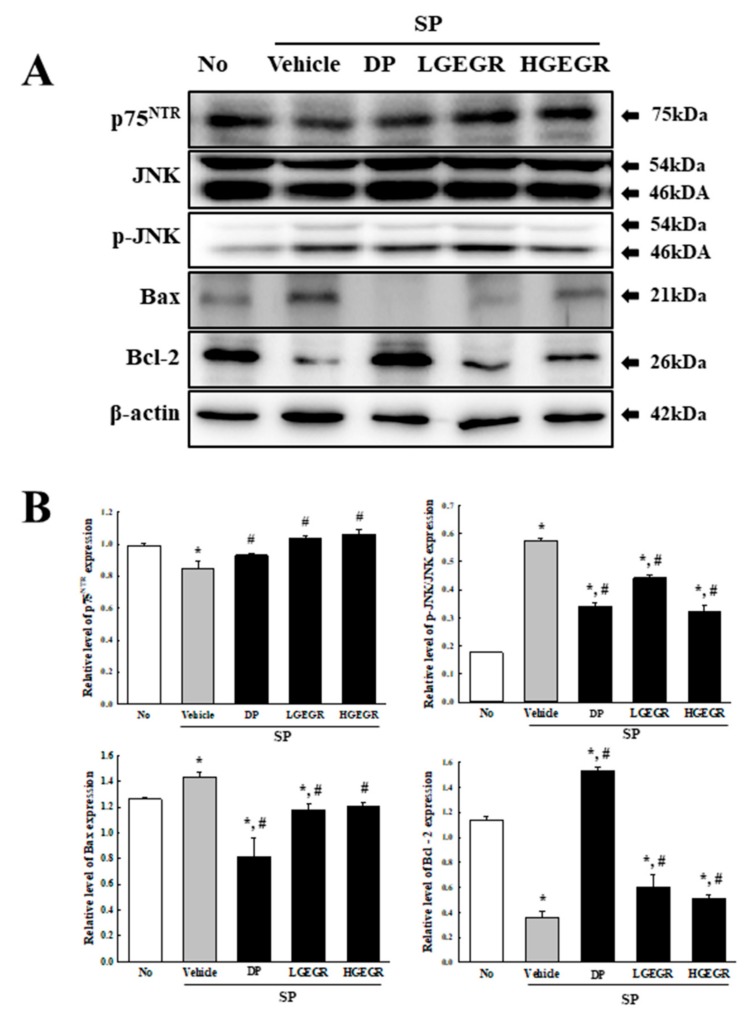
Alteration of the p75^neurotrophin receptor^ (p75^NTR^) receptor signaling pathway. Total tissue homogenates were prepared from brains of Vehicle or GEGR treated SP-injected mice as described in Materials and Methods. Total protein (50 μg per sample) was immunoblotted with p75^NTR^, JNK, p-JNK, Bax, Bcl-2, or β-actin antibodies. After determining individual band intensities using an imaging densitometer, the relative levels of the proteins were based on the intensity of actin. Three samples were assayed in duplicate by western blotting. Data are reported as mean ± SD values. * *p* < 0.05 compared with the No treated group. ^#^ *p* < 0.05 compared with the SP+Vehicle treated group. Abbreviation: DP, donepezil; GEGR, gallotannin-enriched extract of Galla Rhois; SP, scopolamine.

BDNF supports the survival of neurons and stimulates the growth and differentiation of new neurons and synapses in the hippocampus, cortex, and basal forebrain [25]. Therefore, we investigated whether the protective effects of GEGR on SP-induced memory impairment is linked to the regulation of BDNF secretion and the receptor signaling pathway. To achieve this, alterations in the BDNF concentration and BDNF signaling pathway were measured in the brain of SP+GEGR treated mice. The concentration of BDNF was lower in the SP+Vehicle treated group than the No treated group. This level was significantly protected in the SP+LGEGR and SP+HGEGR treated groups as compared to the SP+Vehicle treated group (Figure 4). Furthermore, BDNF secreted from the brains of SP+GEGR treated mice groups transduced the signals into the cytosol by binding two types of BDNF receptors located on the target cell membrane. Analysis for TrkB and p75^NTR^ receptor signaling pathway showed significant alterations in the expression levels of downstream members under their receptors. The TrkB level was remarkably protected in the brain of the SP+DP and SP+GEGR treated group, although it was lower in the SP+Vehicle treated group as compared to the No treated group. A similar protection pattern was observed on the phosphorylation level of PI3K among TrkB downstream members, although the phosphorylation level of ERK remained constant (Figure 5). Similarly, the decreased expression level of p75^NTR^ in the SP+Vehicle treated group was protected after GEGR treatment (Figure 6). These protections were observed in the downstream signaling members. The levels of JNK phosphorylation, Bax, and Bcl-2 expression were significantly protected in the SP+GEGR treated group compared to the SP+Vehicle treated group (Figure 6). These results indicate that the protective effects of GEGR on SP-induced memory impairment is associated with the induction of significant changes in BDNF secretion and TrkB/p75^NTR^ receptor signaling pathway.

### 3.5. Effects of GEGR on the Survival and Function of Neuronal Cells

**Figure 7:**
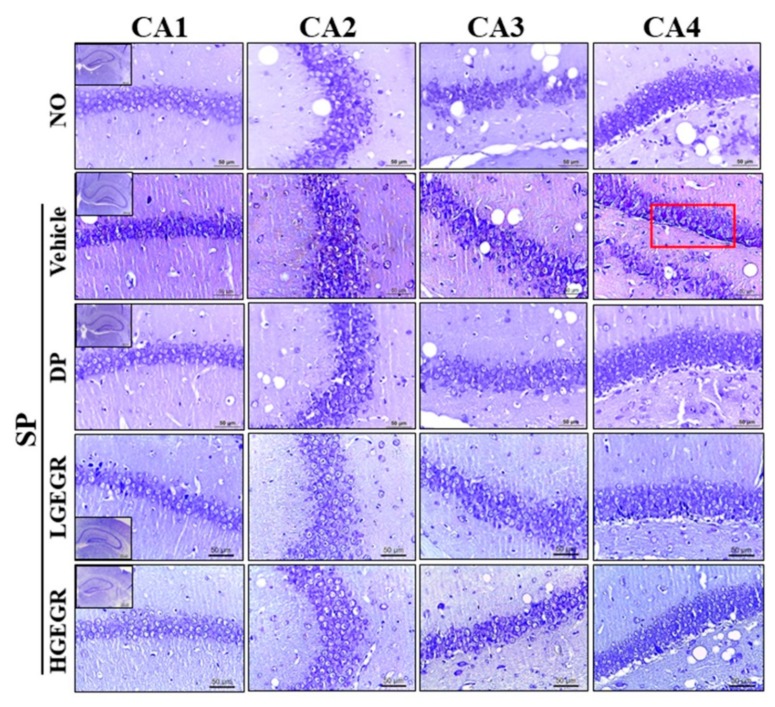
Observation of Nissl-stained neurons in the hippocampus of mice. After SP injection into mice pretreated with GEGR for three weeks, brain tissues were collected from each group and histological changes were assessed as described in Materials and Methods. Slides bearing sections of brain tissue were stained with Nissl and then observed at 400× magnification. Abbreviation: DP, donepezil; GEGR, gallotannin-enriched extract of Galla Rhois; SP, scopolamine; CA, cornu ammonis.

**Figure 8:**
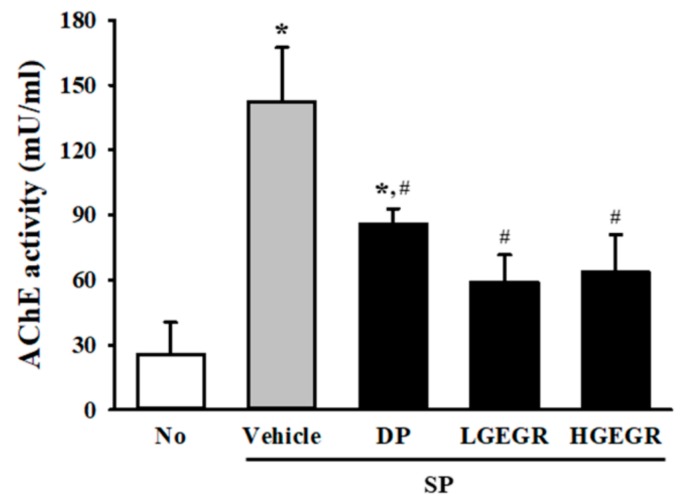
Measurement of acetylcholinesterase (AChE) activity in SP+GEGR treated mice. After SP treatment, AChE activity was measured in homogenates of brain tissues collected from mice in each group by using an acetylcholinesterase assay kit. This assay can detect as little as 0.1 mU AChE in a 100 µL assay volume (1 mU/mL). Data are reported as mean ± SD values. * *p* < 0.05 compared with the No treated group. ^#^ *p* < 0.05 compared with the SP+Vehicle treated group. Abbreviation: DP, donepezil; GEGR, gallotannin-enriched extract of Galla Rhois; SP, scopolamine.

Next, we investigated whether GEGR pretreatment affects the survival and function of neuronal cells in the brain. To achieve this, the number of dead neuronal cells and AChE activity were measured in the SP-induced memory impairment mice after exposure to GEGR. Compared to the No treated group mice, larger numbers of dead neuronal cells were observed in the granular cell layer of the dentate gyrus in the SP+Vehicle treated mice. However, these numbers were remarkably declined in the SP+GEGR treated groups, relative to the SP+Vehicle treated group (Figure 7). There was a similar pattern shown for AChE activity, with the SP+Vehicle treated group showing a higher level (5.36 times) of AChE activity than the No treated group, which were remarkably decreased in the SP+GEGR treated groups (Figure 8). These findings suggest that GEGR pretreatment promotes the survival and function of neuronal cells in the specific region of the brain in the SP-induced memory impairment model.

### 3.6. Effects of GEGR on the Regulation of Inflammatory Cytokines

**Figure 9:**
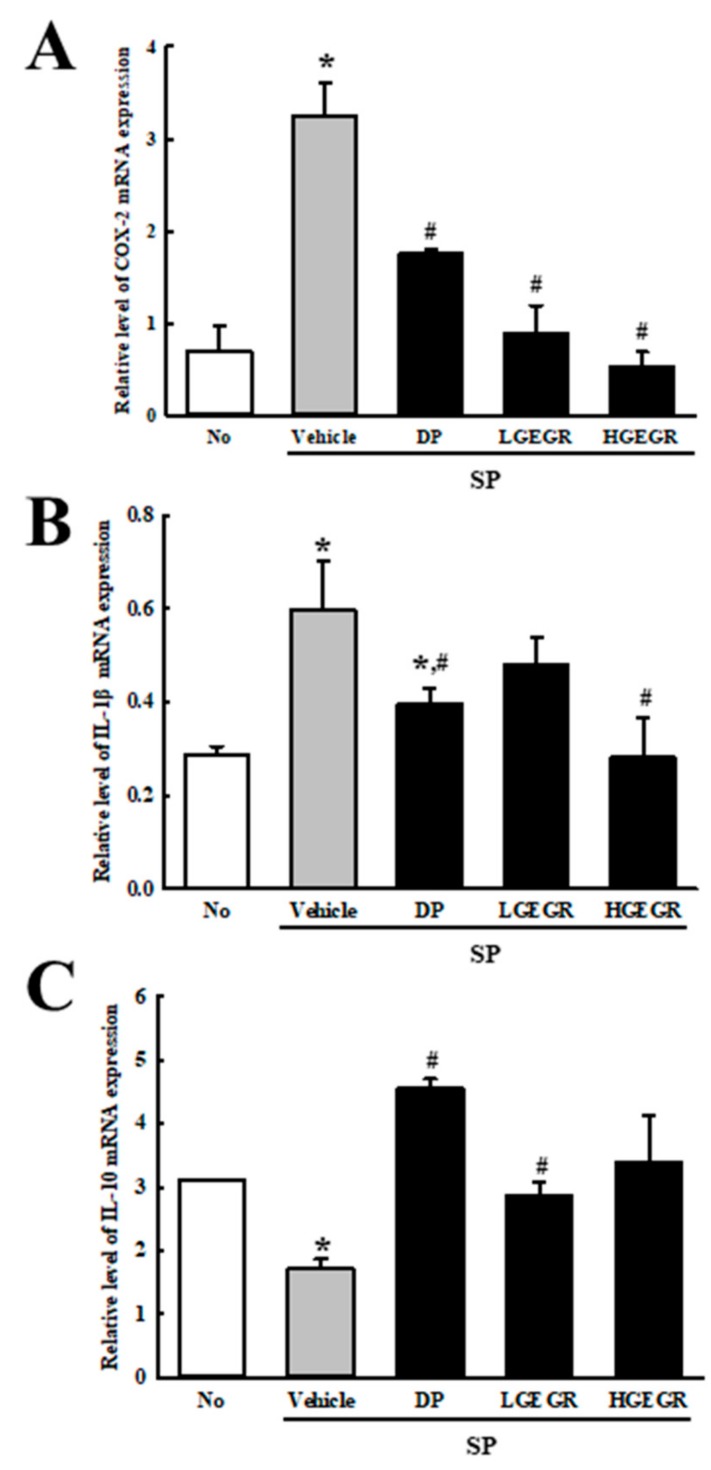
Measurement of inflammatory cytokines and their mediators in SP+GEGR treated mice. The levels of COX-2 (**A**), IL-1β (**B**), and IL-10 (**C**) transcripts in the total mRNA of brain were measured by quantitative real time-PCR analyses using specific primers. The mRNA level of each gene was calculated based on the intensity of actin as an endogenous control. Three samples were assayed in duplicate by qRT-PCR analyses. The values of data represent the mean ± SD. * *p* < 0.05 compared with the No treated group. ^#^ *p* < 0.05 compared with the SP+Vehicle treated group. Abbreviation: DP, donepezil; GEGR, gallotannin-enriched extract of Galla Rhois; SP, scopolamine.

Finally, we examined the role of GEGR on the regulation of inflammatory response during SP-induced memory impairment. To achieve this, alteration on transcripts level of inflammatory cytokine and their mediators were measured in the SP-induced memory impairment mice after exposure to GEGR. The levels of COX-2 transcripts were higher in the SP+Vehicle treated group than in the No group, but significantly decreased in the SP+GEGR treated groups (Figure 9A). Furthermore, a similar pattern was measured on the transcription levels of pro- and anti-inflammatory cytokines. The enhanced or declined transcription levels of IL-1β and IL-10 were significantly inhibited in SP+GEGR treated group (Figure 9B,C). Taken together, these results indicate that the neuroprotective effect of GEGR is associated with the inhibition of inflammatory responses in the brain tissue of SP-induced memory impairment.

## 4. Discussion

Tannins are considered as strong antioxidants and free radical scavengers among the various plant-derived polyphenols, widely found in many herbaceous and woody plants including fruits, sorghum, millets, barley, beans, tea, wine, and berries [26,27]. Especially, they are known to exert beneficial effects in human health since tannins contain many hydroxyl and other functional groups which cross-link to various proteins and macromolecules [28,29]. Therefore, great attention has recently been focused on several tannins, tannin-related extracts, and tannin-containing natural products as novel therapeutic drugs for the treatment of AD due to their significant potential to possibly improve similar diseases [8,10]. In an effort to identify novel drugs for the treatment of AD, we investigated the regulation mechanism of memory retention, neuronal cell survival, and BDNF-related signaling pathway during the neuroprotective effects of GEGR in SP-induced cognitive impaired Korl:ICR mice. The results of the present study clearly demonstrate that GEGR induces neuroprotective effects in the brain, including the protection of cognitive impairment, dysregulation of BDNF-related signaling pathway, and neuronal cell dysfunction induced by SP injection. Furthermore, we believe our data is additional evidence to demonstrate that tannins with high antioxidant activity are capable of suppressing the AChE activity and neuronal cell death, as well as recover the BDNF receptor signaling pathway while effectively alleviating AD.

Several studies have investigated the ability of tannins and tannin-containing natural products to ameliorate memory impairment in various model animals with AD-like phenotypes. The treatment of some tannins (including corilagin, punicalagin, and raisin) ameliorated the memory impairment in radiation-induced brain injury (RIBI), LPS-induced neuroinflammation resulting in memory impairment, and aluminum chloride injected model [30,31,32]. Similar memory enhancing effects were induced after treatment with tannin-containing natural products. Ethyl acetate extract (EAE) of leaves of *Ugni molinae* Turcz prevented the deterioration of memory capacity in APPswe/PS1dE9 Tg mice, while the green *Ocimum basilicum* hydroalcoholic extract increased memory retention [33,34]. The hydroalcoholic extract from *Emblica officinalis* showed the beneficial effects on SP-induced memory impairment in Swiss albino mice [35]. However, little is known about the direct effects of GEGR on SP-induced memory impairment using animal models expressing AD pathological features. The results of our study are in agreement with previous reports, although there are few differences in the treatment conditions and enhancement rates. Furthermore, our results are the first to demonstrate that SP-induced memory impairment in Korl:ICR mice is effectively enhanced by administration of GEGR for three weeks.

Meanwhile, ROS has been defined as reactive chemical species containing oxygen and included superoxide, hydroxyl radical, H_2_O_2_, and singlet oxygen [36]. It widely used one of key indicators for oxidative stress because ROS mediates various cellular responses such as proliferation, differentiation, apoptosis, and death at different levels [37]. Many types of methods have developed to measure ROS using fluorescent and chemiluminescent probe, spectrophotometry, and chromatography [38]. Among these, DCFH-DA generally used for direct measurement of intracellular redox states [39]. Therefore, in this study, the ROS level in B35 cells and brain tissue was detected based on the oxidation of DCFH-DA. However, the application of this method has some limitations including an incomplete reflection of any specific free radicals, the formation of probe radical intermediate, and the promotion of DCFH oxidation in the absence of H_2_O_2_ [40,41,42,43].

Neurotropic factors have pivotal roles in the mechanisms underlying neuronal cell survival, differentiation of dendritic branching and dendritic spine morphology, synaptic plasticity and cell apoptosis [44]. This neurotrophin family includes BDNF, nerve growth factors (NGF), and neurotrophins. Of these, BDNF appears essential for the molecular mechanism in neural development, cell survival, and synaptic plasticity [45]. It binds one or more of the Trk receptor and p75^NTR^ receptor linked to several intracellular signal transduction pathways, including PLC-γ1 (phospholipase C), PI3K, MAPK, NF-κB, and Jun kinase [46,47,48]. BDNF concentration and their receptor signaling pathways were significantly facilitated with GEGR treatment in our study. Especially, the SP+GEGR treated groups show high levels of BDNF and the recovery of TrkB receptor and p75^NTR^ receptor signaling pathways in western blot analyses. Similar results have been reported in previous studies investigating the efficacy of tannin-containing natural products. The plant-derived flavanol (-)epicatechin increased the concentration of BDNF and pro-BDNF in bromodeoxyuridine-injected C57BL/6 mice, while the pine needle extract containing 10.15% tannin stimulated the expression of BDNF and pCREB in the SP-induced memory impaired model [49,50]. Moreover, persimmon fraction containing very high levels of soluble tannins suppresses the expression and phosphorylation of several downstream members of BDNF receptor signaling pathway in trimethyltin chloride (TMT)-treated ICR mice [51]. Our data suggests that the ameliorating effects of GEGR on memory impairment could be explained, at least in part, by their protection of BDNF secretion and their receptor signaling pathway within the brain. However, more study on the analysis for pro-BDNF and BDNF ratio are necessary to clearly understand the role of pro-BDNF and BDNF signaling pathway during the protection effect of GEGR. Actually, this ratio can provide key data to understand the GEGR action mechanism on the BDNF regulation pathway because pro-BDNF and BDNF induce opposite biological functions through regulating two different receptor systems [52]. Furthermore, a significant decrease of the pro-BDNF and BDNF ratio in the hippocampus can cause the depression-like behaviors and alterations of CA1 pyramidal neurons [53].

AChE in the brain influences the alteration of neuronal excitability, the regulation of synaptic transmission, the induction of synaptic plasticity, and coordination of the firing of neurons during cholinergic regulation [54]. Therefore, these factors are considered as key regulation factors to evaluate the neuroprotective effects of various natural products. In this study, we investigated the therapeutic effects of GEGR on the pathological symptoms of the AD model through the suppression of AChE activity. Our results are the first to show that AChE activity in the SP-induced memory impaired model are effectively suppressed by administration of GEGR for three weeks. Moreover, similar suppression abilities of tannin-related products have been investigated in several previous studies, although with varying rates. Decreased AChE activity has been reported in Wistar rats treated with grape seed proanthocyanidin extracts (GSPE), aged impaired rats treated with procyanidins of lotus seedpod, SP-induced amnesia model treated with *Emblica officcinalis* extract, and chronic mild stress model treated with *Terminalia catappa* leaf extract [35,55,56,57]. Therefore, our study indicates a possibility that GEGR containing a high concentration of tannin may be considered a potential AChE inhibitor to treat AD, although additional research is required.

## 5. Conclusions

In summary, the present study investigates the potential therapeutic action of GEGR for AD treatment, particularly the attenuation of memory impairment and neuronal dysfunction in SP-treated mice. Our study demonstrates that GEGR significantly decreases the AChE activity and improves the survival and function of neuronal cells in the brains of SP-treated mice through the stimulation of BDNF activity and suppression of oxidative stress.
